# Understanding gold toxicity in aerobically-grown *Escherichia coli*

**DOI:** 10.1186/s40659-020-00292-5

**Published:** 2020-06-08

**Authors:** C. Muñoz-Villagrán, F. Contreras, F. Cornejo, M. Figueroa, D. Valenzuela-Bezanilla, R. Luraschi, C. Reinoso, J. Rivas-Pardo, C. Vásquez, M. Castro, F. Arenas

**Affiliations:** 1 Laboratorio Microbiología Molecular, Departamento de Biología, Facultad de Química y Biología, Universidad de Santiago de Chile, Santiago, Chile; 2 Laboratorio de Microbiología Aplicada, Departamento de Ciencias Básicas, Facultad de Ciencias, Universidad Santo Tomás, Sede Santiago, Chile; 3 Laboratorio de Biología estructural, Centro de Genómica y Bioinformática, Universidad Mayor, Santiago, Chile

**Keywords:** Gold(III), Toxicity, Resistance, *E. coli*, Aerobic, Anaerobic

## Abstract

**Background:**

There is an emerging field to put into practice new strategies for developing molecules with antimicrobial properties. In this line, several metals and metalloids are currently being used for these purposes, although their cellular effect(s) or target(s) in a particular organism are still unknown. Here we aimed to investigate and analyze Au^3+^ toxicity through a combination of biochemical and molecular approaches.

**Results:**

We found that Au^3+^ triggers a major oxidative unbalance in *Escherichia coli*, characterized by decreased intracellular thiol levels, increased superoxide concentration, as well as by an augmented production of the antioxidant enzymes superoxide dismutase and catalase. Because ROS production is, in some cases, associated with metal reduction and the concomitant generation of gold-containing nanostructures (AuNS), this possibility was evaluated in vivo and in vitro.

**Conclusions:**

Au^3+^ is toxic for *E. coli* because it triggers an unbalance of the bacterium’s oxidative status. This was demonstrated by using oxidative stress dyes and antioxidant chemicals as well as gene reporters, RSH concentrations and AuNS generation.

## Background

In addition to carbon, hydrogen, oxygen, and nitrogen, biomolecules are made of a number of other elements such as iron, calcium and magnesium, among others [1]. Some of them are critical for several biochemical processes and form part of the cell membrane, nucleic acids as well as protein structure [2]. Metals like iron are so fundamental for life that they are often referred to as essential [3]. The list of essential elements includes also Na, Mg, K, Ca, V, Cr, Mn, Fe, Co, Ni, Cu, Zn, Se and Mo [2]. On the other hand, non-essential metals—i.e., those that do not display known biological roles in living organisms—, include Ag, Hg, Te, and Au [2], and are actually extremely toxic for microorganisms [1].

Given their toxicity, a number of these non-essential metals are currently being used as antimicrobial compounds by incorporating them on surfaces and coatings medical and pharmaceutical equipment [4]. Particularly, and since silver, gold, copper and titanium exhibit physicochemical characteristics favoring their antimicrobial activity, they are nowadays usually included in several nanomaterials [5]. The toxicity of some metals and metalloids such as chromate and tellurite is related to the generation of reactive oxygen species (ROS) [6–10], which then damage key cell components and affect bacterial growth [11].

Gold (III) is toxic for *E. coli*, displaying a minimal inhibitory concentration (MIC) around 20 µM, twice that of the extremely toxic Hg(I) [1]. In a more recent article, Nam and coworkers showed that Au^3+^ altered significantly the growth of *E. coli*, *Bacillus subtilis* and other aquatic microorganisms [12]. Although the specific mechanism of gold toxicity for *E. coli* has not been elucidated yet, given that Au^3+^ belongs to the group of soft metal ions [13], it is probable that it interacts with soft bases such as thiols and/or other cell targets containing soft base groups [2]. Since one of the main reduced thiol targets is glutathione (GSH) [13], Au^3+^ uptake could generate an intracellular redox unbalance that ultimately affects the viability of the microorganism [14]. Transcriptomic microarray experiments were conducted to assess the response of *Cupriavidus metallidurans* CH34 to aqueous Au(III)-complexes which appear to be toxic because they become oxidative once inside the cell [15, 16].

Currently, nanostructures (NS) containing some of these non-essential metals are used in diverse applications such as water treatment, photocatalysis, optics and therapeutic procedures, among others [5, 17]. In particular, gold nanostructures (AuNS) behave as potent antimicrobial agents against multidrug resistant Gram-negative and Gram-positive bacteria [18]. In general, it has been suggested that AuNS would be toxic because they can generate ROS, similar to that seen in many other antibacterial materials such as ZnO, TiO_2_ and Ag nanoparticles (NPs) [17, 19, 20]. Other targets depend on the route of gold(III) complexes synthesis or composition; for instance, AuNPs covered with organic molecules such as 4,6-diamino-2-pyrimidinethiol produce i) a change in membrane potential that limits ATPase activity and ii) inhibit tRNA binding to the ribosome, thus leading to a collapse of several biological processes without ROS production [21]. In spite of this evidence, specific mechanism(s) by which Au^3+^ triggers cell toxicity are still unanswered.

In this work, we aimed to shed light on Au^3+^ antimicrobial activity. By combining ROS-sensitive probes, oxygen scavengers, transcriptional induction analysis and intracellular thiol concentration determinations, we found that *E. coli* exposure to Au^3+^ results in the generation of an oxidative unbalance characterized by increased ROS levels, overproduction of antioxidant enzymes, decreased levels of reduced thiols, and in vivo and in vitro generation of AuNS. These findings represent an initial attempt to elucidate the molecular basis of bacterial Au^3+^ toxicity.

## Results

**Fig. 1:**
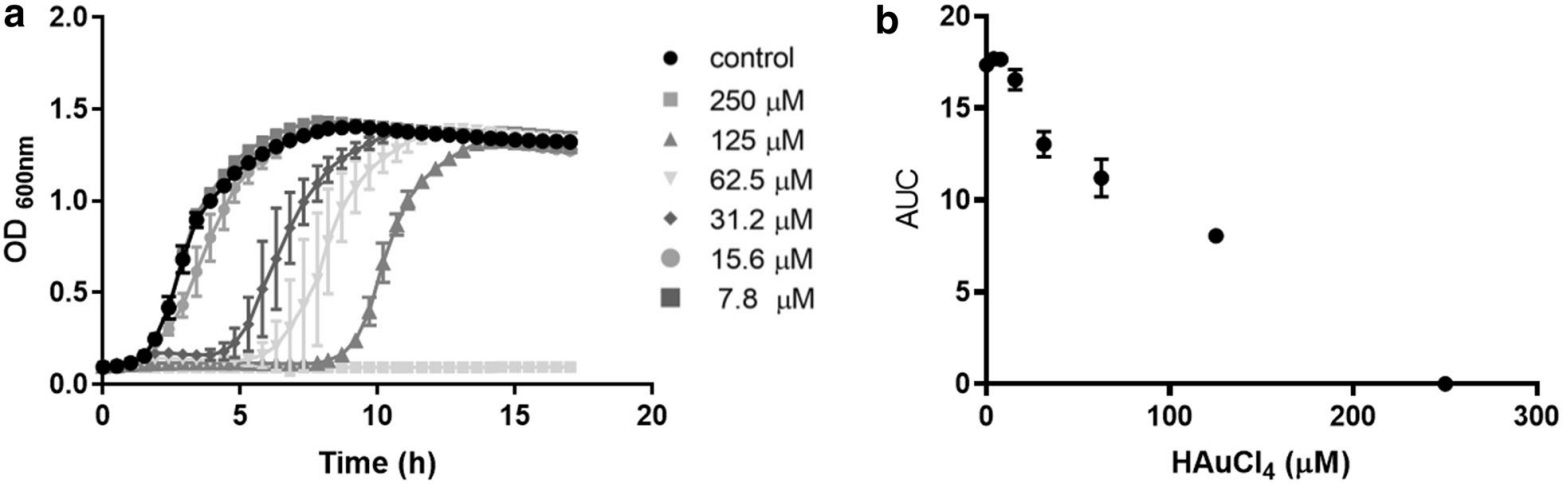
*E. coli* susceptibility to HAuCl_4_. **a***E. coli* growth in the presence of different gold concentrations under aerobic conditions. **b** Relationship of the area under the curve (AUC) and HAuCl_4_ concentration

A number of tests including susceptibility assays, growth curves, growth inhibition areas and MIC were used to characterize the toxicity triggered by Au^3+^ in *E. coli*. Under aerobic conditions, the Au^3+^ MIC was 250 μM and growth inhibition area was 1.12 ± 0.02 cm^2^. Negligible effects on cell growth were observed when *E. coli* was exposed to lower Au^3+^ concentrations (7.8–15.6 µM). Nevertheless, when the metal concentration was raised to 31.2–125 µM, the lag phase was extended compared to that exhibited by untreated controls. No growth was observed at Au^3+^ concentrations above 250 µM (Fig. 1a). To assess the impact of the metal on *E. coli* growth, the Area Under Curve (AUC) of the growth curve at each concentration tested was calculated. Figure 1b shows a dose–response relationship between HAuCl_4_ concentration and AUC, indicating that at Au^3+^ concentrations above 250 µM growth is severely inhibited.

**Fig. 2:**
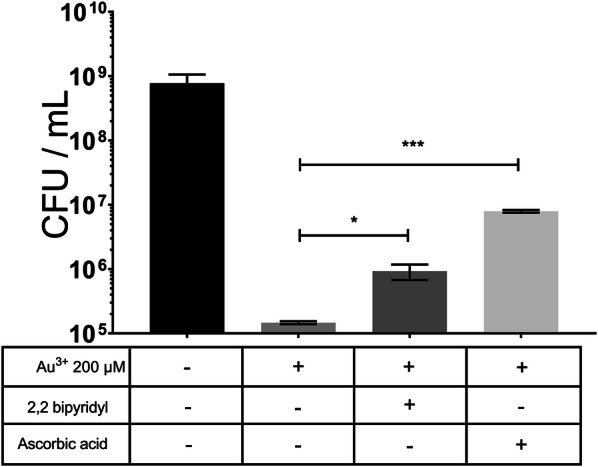
Effects of ROS scavengers on the viability of gold-exposed *E. coli*. Viability of cells treated with 200 µM HAuCl_4_ was assessed in the absence (control) and presence of the toxicant and the scavengers 2,2 bipyridil (1 mM) and ascorbic acid (10 mM). Data represent the average of 3 independent trials ± SD

We hypothesized that the initial lag observed with HAuCl_4_ above 15.6 µM and below 250 µM could be due to the establishment of an oxidative stress status, a phenomenon well documented for other metal(loid)s [8, 22, 23]. Then, cell viability was determined in the presence of two ROS scavengers: ascorbic acid [24] and 2,2´-Bipyridyl (iron chelator that prevents hydroxyl radical production) [25]. Figure 2 shows the effect of both scavengers on cells exposed to 200 µM HAuCl_4_. The presence of either ascorbate or 2,2´-Bipyridyl reverted, although not completely, the toxic effects of Au^3+^ regarding untreated controls. These results suggest that, at least in part, Au^3+^ toxicity is ROS-mediated.

**Fig. 3:**
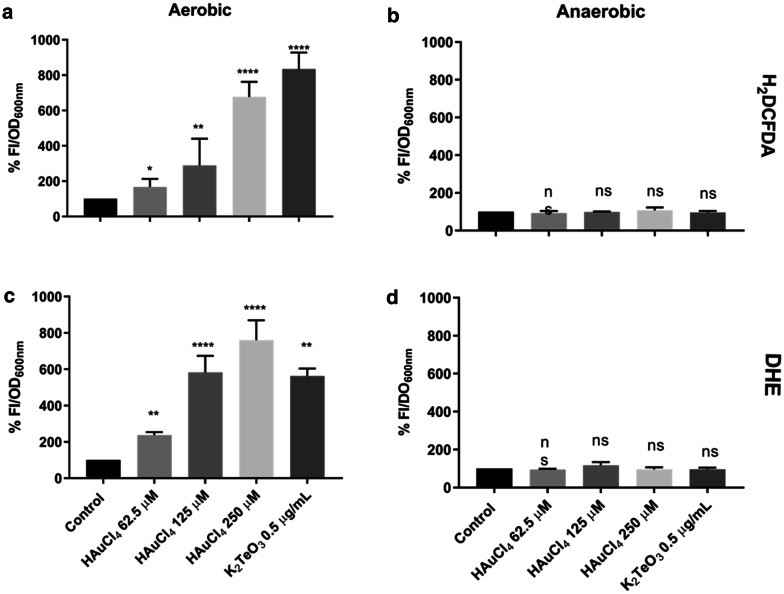
Reactive Oxygen Species in HAuCl_4_-exposed *E. coli*. Total ROS and superoxide levels were assessed using H_2_DCFDA (**a, b**) and DHE (**c, d**) in gold-treated *E. coli*. The assays were conducted under aerobic (**a, c**) and anaerobic (**b, d**) conditions. The percentage in relation to untreated controls (100%) of the fluorescence intensity normalized by protein production was plotted. Data represent the average of 3 independent assays ± SD. Statistical significance was according to section Data analysis

ROS generation by HAuCl_4_ was assessed using the fluorescent probes H_2_DCFDA and DHE, which allow determining total ROS and superoxide, respectively. Figure 3 shows ROS formation in *E. coli* exposed to HAuCl_4_ under aerobic and anaerobic conditions. Tellurite-mediated ROS generation [8, 26] was included for comparison. As expected, total ROS as well as superoxide levels increased with increasing Au^3+^concentrations (Fig. 3a, c). In turn, no changes were detected in the absence of oxygen (Fig. 3b, d). The probe altogether with the metal did not generate increased fluorescence (cell-free control, not shown).

**Fig. 4:**
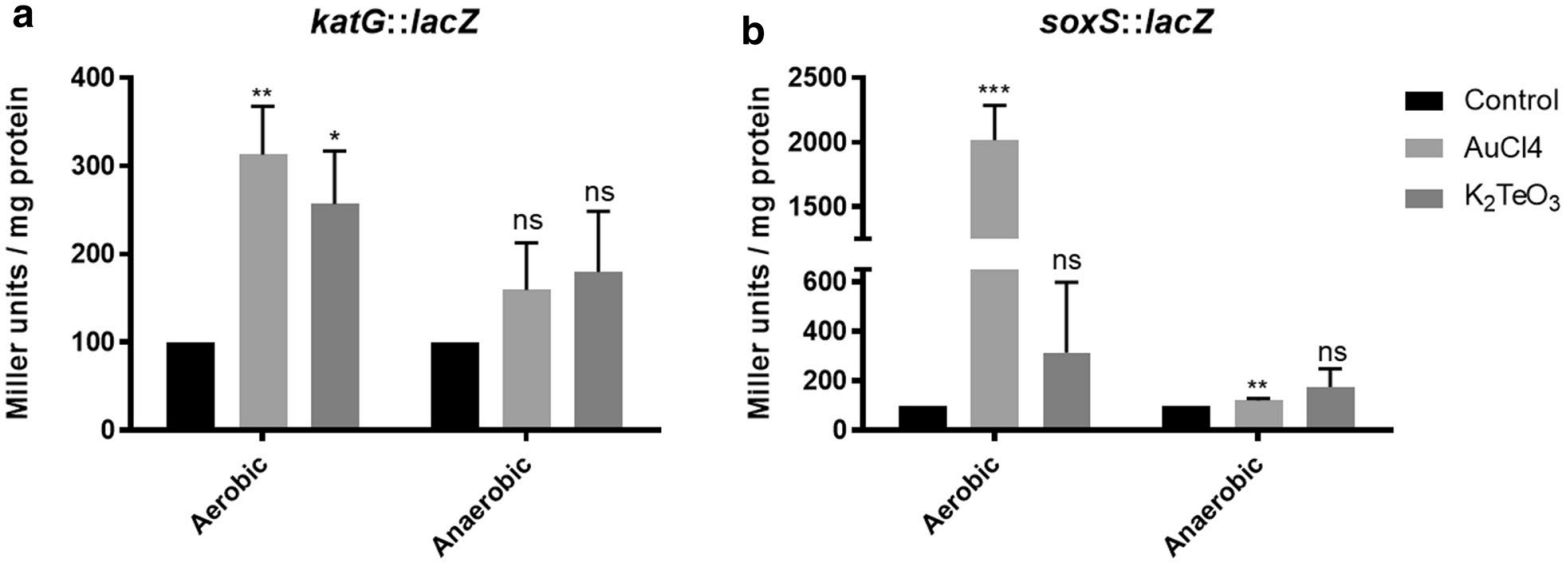
*katG* and *soxS* expression in HAuCl_4_ treated-*E. coli*. Gene expression was monitored using *lac*Z fusions in *E. coli* GS022 (*katG*::*lacZ*) (**a**) and SP11 (*soxS*::*lacZ*) (**b**), in absence (control) or presence of HAuCl_4_ (125 µM) or K_2_TeO_3_ (2 µM). Data represent the average of 3 independent trials ± SD. Statistical significance was according to section Data analysis

Given that Au^3+^ exposure results in increased ROS levels, a transcriptional response was also expected. Such response was investigated using *E. coli* strains GS022 and SP11, which contain the *lacZ* gene downstream the promoters of *katG* and *soxS* genes, respectively [27]. Figure 4 shows that the reporter activity increases upon exposure to 200 µM HAuCl_4_, a change that was observed only under aerobic conditions. The highest induction (200-fold) was observed in SP11 cells (Fig. 4b), while the *katG*::*lacZ* construct was induced only three fold regarding untreated controls (Fig. 4a). Taken together, these results allow speculating that enzymes such as catalase, peroxidase, superoxide dismutase and probably glucose-6-phosphate dehydrogenase are overproduced in response to HAuCl_4_.

**Fig. 5:**
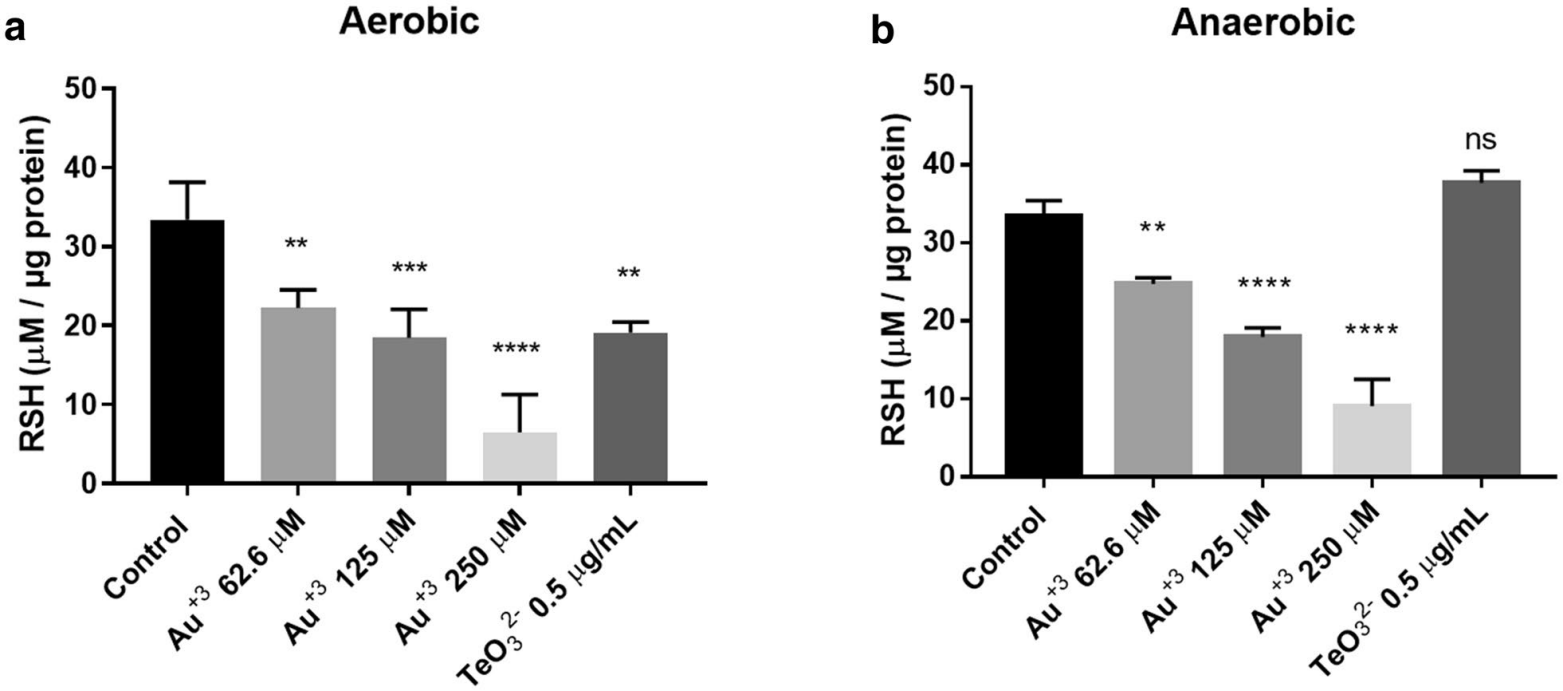
Intracellular reduced thiol levels in HAuCl_4_ treated-*E. coli*. The assays were carried out under aerobic (**a**) and anaerobic (**b**) conditions in the presence of different concentrations of Au^3+^ or tellurite (positive control). Data represent the average of 3 independent assays ± SD

In general, oxidative stress alters the equilibrium among some cellular components; in this line, the thiol reactive dye DTNB was used to assess the intracellular redox unbalance triggered by the toxicant. Tellurite and menadione, two compounds previously shown to affect intracellular thiol (RSH) levels were used as positive controls [8]. As expected, tellurite caused a change in thiol levels only under aerobic conditions, while menadione decreased RSH levels both in aerobiosis and anaerobiosis. Au^3+^ treatment resulted in a dose-dependent decrease of the RSH pool (Fig. 5), a result that matchs our initial observations regarding HAuCl_4_ toxicity in an oxygen-free environment (Additional file 1: Figure S1).

**Fig. 6:**
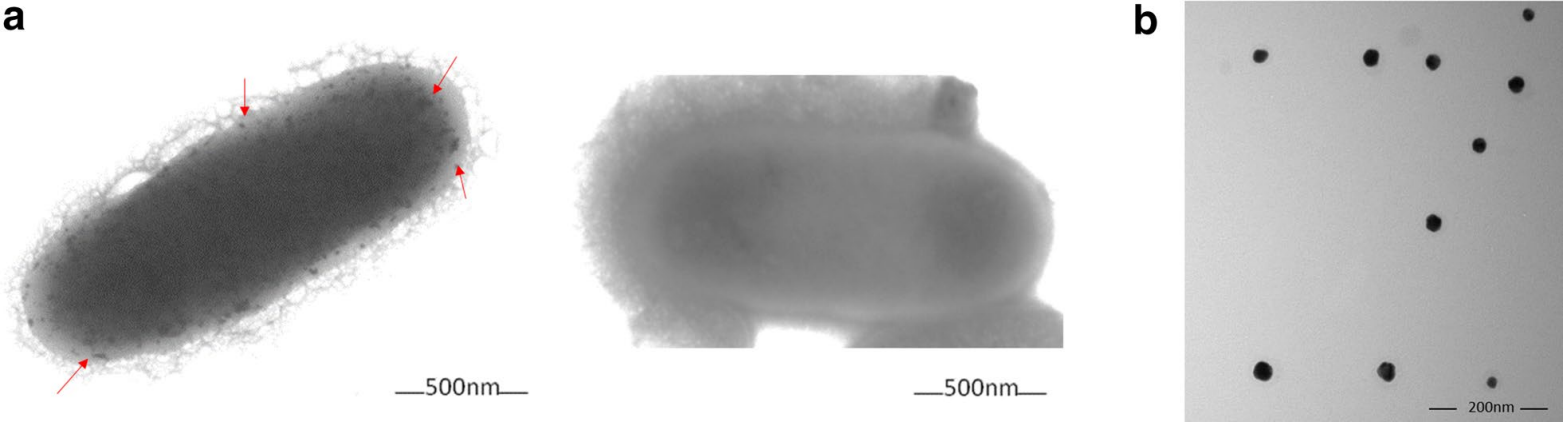
*In vivo* and in vitro synthesis of AuNS. **a** Electron micrographs of *E. coli* exposed to ¼ of the MIC to HAuCl_4_ in aerobiosis (left) and untreated (right). Arrows indicate AuNS. **b** Electron micrographs of in vitro synthesized AuNS by *E. coli* crude extracts (aerobic conditions)

Finally, it was analyzed if HAuCl_4_-mediated ROS generation in *E. coli* is accompanied by a concomitant generation of AuNS which eventually would decrease metal toxicity. Figure 6a shows that *E. coli* forms AuNSs that accumulate homogeneously within the bacteria in the presence of 1 mM gold (III). AuNSs were also synthesized in vitro using cell-free crude extract (Fig. 6b), which showed a spheroidal shape and an average size of 20–30 nm.

## Discussion

Some metals are essential in trace amounts for the functioning of living organisms; however, most of them become toxic at higher concentrations [1]. Part of the toxicity exhibited by heavy metal cations such as Hg^+^, Cd^+^ and Ag^+^ occurs mainly because of their trend to bind cellular thiol groups like glutathione, especially in Gram negative bacteria [28]. Nevertheless, it has been also shown that their toxicity is related to the generation of an oxidative stress status in the cell [2, 9], thus affecting other cell targets such as thiol content [29] and heme biosynthesis [30].

Although harmful effects of gold for microorganisms are partially known [1], detailed gold(III) toxicity studies are still scarce. Some examples include the synthesis of gold nanostructures, which are being currently used as antimicrobial agents [31] and in other biomedical applications [32, 33].

Susceptibility assays showed that gold effects on *E. coli* growth are proportional to metal concentration and that the lag phase was affected in the presence of 31–125 µM HAuCl_4_ (Fig. 1a, b). The gold minimal inhibitory concentration for *E. coli* was 250 µM, where, as expected, a total inhibition of the bacterial growth was observed (Fig. 1).

Since several metals are toxic because of ROS production, oxidative damage generation upon gold exposure was evaluated under aerobic conditions in the presence of the ROS scavengers 2,2´-Bipyridyl and ascorbic acid [21]. Figure 2 shows that 2,2´-Bipyridyl improved significantly the growth of *E. coli* exposed to Au^3+^, probably because of a decreased formation of hydroxyl radicals. Likewise, the presence of ascorbic acid favored *E. coli* growth by more than two log units. In this line, it has been shown that the low redox potential of ascorbate protects against increased metal-induced superoxide generation [34]. While ascorbic acid and 2,2´-Bipyridyl have been used as scavengers of ROS to evaluate the antioxidant effect of certain compounds, in other studies ascorbate is used as Au(III)-reducing agent and 2,2-Bipyridyl to synthesize nanoparticles [35, 36]. It is possible that there is an interaction of these molecules with Au(III), which would decrease metal bioavailability and therefore toxicity (Fig. 2). Formation of gold NS are carried out under defined conditions, for example, high ascorbate concentrations allow rapid gold reduction at acid pH [35], which does not occur in our conditions. Furthermore, nanoparticles and bipyridyl form complexes that are adducts linked by coordination with HAuCl_4_*3H_2_O and derivatives of 6-benzyl-2,2′-Bypiridine in ethanol solution [37]. Then, to rule out the putative interaction between Au(III) and these antioxidant molecules, experiments were repeated adding this time a pre-incubation step of cells grown up to OD ~ 0.4 nm for 30 min; cells were then washed with fresh medium and treated with 0.2 mM Au(III) for 15 min. Results indicated that the antioxidants generate a protective effect against toxicant-generated ROS (Additional file 2: Figure S2).

On the other hand, ROS generation was assessed using the fluorescent probes H_2_DCFDA and DHE, which detect total ROS [38] and superoxide [39], respectively. Cells treated with Au^3+^ showed increased fluorescence that was proportional to toxicant concentration, thus indicating that Au(III) indirectly produces ROS (Fig. 3). However and as expected, this effect was only observed under aerobic conditions. Particularly, higher fluorescence was observed with the superoxide-detecting probe, suggesting that O_2_^−.^ would be the main ROS generated by Au(III) in *E. coli* (Fig. 3c).

Because of the above results, the cell antioxidant response was evaluated. *E. coli* exposure to Au^3+^ also resulted in increased induction of *soxS* and *katG* genes in aerobic conditions, (Fig. 4). Since SoxS activates a group of enzymes that mitigate the effects of superoxide [40], these results support the observation that Au^3+^ generate ROS. Peroxide formed from superoxide dismutation by the enzyme superoxide dismutase [41] is in turn decomposed to H_2_O and O_2_ by KatG, hydroperoxidase I (HPI) and/or catalase.

Since gold -like other soft metals- displays affinity for soft bases such as sulfhydryl groups [2] which could result in a redox unbalance [42], the effect of gold exposure on the level of cell RSH was evaluated. The level of reduced cellular thiols decreased upon gold treatment both in aerobic and anaerobic conditions (Fig. 4). Surprisingly, *E. coli* growth was more affected in the absence of oxygen (Additional file 1: Figure S1); although toxicity of this metal in this case is independent of ROS, it has also been described that it is still toxic in anoxic environments [43]. This interesting anaerobic effect is under investigation in our laboratory.

Finally, one of the putative mechanisms of bacterial response to metal(loid)s is their reduction to the respective elemental state, which has been widely studied [44–47]. Given that *E. coli* crude extracts were able of gold(III) reduction generating a characteristic red precipitate (not shown), the possibility of synthesizing AuNS in vivo and in vitro was explored (Fig. 6). *E. coli* formed AuNS which accumulated homogeneously inside cells, suggesting that AuNS formation by *E. coli* could be consequence of a series of metabolic events in response to HAuCl_4_ exposure.

## Conclusion

Au^3+^ is toxic for *E. coli* because it triggers an unbalance of the bacterium’s oxidative status. This was demonstrated by using oxidative stress dyes and antioxidant chemicals as well as gene reporters, RSH concentrations and AuNS generation.

## Methods

### Strains and growth conditions

*E. coli* BW25113, SP11, and GS022 [27] used in this work were grown in LB medium [48] as previously described [49]. All procedures were carried out at 37 °C under aerobic and, eventually, anaerobic growth conditions. Cells were cultured in thermostabilized orbital shakers; oxygen deprived cultures were conducted inside an anaerobic chamber filled with 100% N_2_ (Coy Lab Products). Inside the Coy chamber a multimode plate reader TECAN equipment was available for anaerobic experiments.

### Growth curves

Overnight cultures were diluted 1:100 with fresh LB medium and incubated in an orbital shaker to OD_600nm_ ~ 0.6. Then, 10 μL were added to 1 mL of fresh LB medium containing different concentrations of HAuCl_4_. Bacterial growth was monitored every 30 min at 600 nm for 18 h using a multimode plate reader (TECAN Infinite M200 Pro). The area under the curve (AUC) [50, 51] was calculated with the R package Growth Curver as described by Sprouffske and Wagner [52].

### Determination of the minimal inhibitory concentration (MIC)

MIC determinations were carried out using serial dilutions (1:2) of a sterile solution of HAuCl_4_ in LB medium in 48-well plates. Subsequently, 10 μL of cultures grown in LB medium to OD_600nm_ ~ 0.6 were added to each well and incubation proceeded with constant shaking at 37 °C. MICs were determined after 24 h of incubation.

### Determination of growth inhibition zones

Overnight cultures were diluted 1:100 with fresh LB medium and incubated with shaking to OD_600nm_ ~ 0.6. After dilution to OD_600nm_ ∼ 0.1, 100 µL were evenly spread on agar LB-plates. After air drying, 10 µl of 50 mM HAuCl_4_ were deposited on sterile filter disks placed on the centers of the plates as described by Contreras et al. [53]. Growth inhibition areas were determined after overnight incubation at 37 °C. To make a correct analysis of the absorbance data obtained in the TECAN plate reader and thus compare the effect on the doubling time, the load capacity and growth rate, the R Growth curver software was used [52]. The AUC (arbitrary unit) metric integrates the information of the parameters K (maximum possible size of the population), r (intrinsic growth rate of the population) and N_0_ (size of the population at the beginning of the curve). These parameters are useful to summarize and compare cell growth dynamics [52], and corroborated that toxicant concentration affects directly the bacterial population.

### Cell viability

Overnight cultures grown in LB medium were diluted (1:100) to OD_600nm_ ∼ 0.4. Then the following treatments were conducted: bacteria were grown in the absence or presence of 200 µM HAuCl_4_, supplemented or not with 1 mM 2,2´-Bipyridyl or 10 mM ascorbic acid. Cultures were treated for 15 min and then serial dilutions were plated on LB/agar. CFU were determined after overnight incubation at 37 °C.

### β-galactosidase assay

*E. coli* SP11 (*soxS*::*lacZ*) and GS022 (*katG*::*lacZ*) were used for stress-promoter activation assays as described by Arenas et al. [27]. Thirty ml of LB medium were inoculated with 300 µl of overnight cultures and grown at 37 °C under aerobic or anaerobic conditions to OD_600nm_ ~ 0.4. Aliquots of 6 mL were treated with HAuCl_4_ (125 µM), K_2_TeO_3_ (2 µM) or without the toxicants for 30 (SP11) and 25 min (GS022), respectively. After incubating on ice for 15 min, OD_600nm_ was determined and cells were sedimented by centrifugation at 13,000× *g* for 3 min. Cell pellets were permeabilized with chloroform (1%) and sodium dodecyl sulfate (SDS 0.1%), and suspended in 1.5 mL of previously chilled buffer Z (40 mM Na_2_HPO_4_ H_2_O; 60 mM NaH_2_PO_4_ 7 H_2_O, pH 7.5 that contained 10 mM KCl, 1 mM MgSO_4_ and 50 mM β-mercaptoethanol). Assays were carried out in triplicate using the chromogenic substrate O-nitrophenyl-β-d-galactopyranoside (ONPG) according to the method described by Miller [14]. The activity was expressed in Miller units [1000 x ((1.75 × OD_550_)−OD_420_)/OD_600_ x t x V/mg protein].

### ROS determination

In general, aerobically- and anaerobically-generated ROS were assessed using the oxidation-sensitive probe 2′,7′-dihydrodichlorofluorescein diacetate [38]. Briefly, cells grown aerobically or anaerobically in LB medium to OD_600nm_ ∼ 0.4 were exposed for 15 min to HAuCl_4_ (250; 125 or 62.5 µM) or to K_2_TeO_3_ (2 µM). Then, cultures were centrifuged, washed with 50 mM potassium phosphate buffer pH 7.0 and incubated for 30 min in the same buffer containing the probe in the dark (40 µM final concentration) to 37 °C. Cells were subsequently washed and pellets suspended with 1 mL of the same buffer; fluorescence intensity was determined in a multi-well plate reader (TECAN Infinite^®^ M200 Pro) using excitation and emission wavelengths of 490 and 527 nm, respectively. Emission values were normalized by the optical density at 600_nm_.

Superoxide generation was assessed as follows. *E. coli* was grown for 30 min as above. After centrifuging and washing with 50 mM potassium phosphate buffer pH 7.0 and incubating in the dark for 15 min with 40 µM dihydroethidine (DHE) to 37 °C, cells were washed, pellets suspended with 1 mL of the same buffer and fluorescence intensity determined using 200 µL of the culture in a multi-well plate reader (TECAN Infinite^®^ M200 Pro, excitation 490_nm_, emission 625 nm). Emission values were normalized as above.

### Determination of reduced thiol concentration

To quantify intracellular thiol content, overnight grown *E. coli* cultures (aerobically or anaerobically) were diluted 1:100 with LB medium and incubated at 37 °C with shaking at 150 rpm to OD_600nm_ ~ 0.5. Then, were treated with HAuCl_4_ (250; 125 or 62.5 µM) or K_2_TeO_3_ (2 µM); 500 µL aliquots were taken after 15 min and centrifuged at 10,000x*g* for 5 min. Sediments were suspended in 1 mL of a solution that contained 5 mM EDTA, 0.1% SDS, 0.1 mM DTNB and 50 mM Tris–HCl buffer pH 8.0. The suspension was incubated for 30 min at 37 °C and subsequently centrifuged at 10,000x*g* for 10 min. Supernatants were recovered and the absorbance at 412 nm was determined in a multi-well plate reader. RSH concentration (µM) was calculated using calibration curves constructed with GSH standards (0–200 µM). RSH values were normalized by the protein concentration.

### In vivo and in vitro synthesis of gold nanostructures

For synthesizing gold nanostructures in vivo*, E. coli* were grown to exponential phase (OD_600nm_ ∼ 0.5), treated with ¼ of the Au^3+^ MIC, incubated for 4 h and centrifuged at 9000x*g* for 10 min. The bacterial pellet was observed by Transmission Electron Microscopy (TEM) in a Philips Tecnai 12 Bio Doble TEM equipment operating at 200 kV as described by Correa-Llantén et al. [44].

Formation of AuNS in vitro was carried out using cell-free extracts (in 20 mM phosphate buffer containing 100 μg/mL of protein) and incubated overnight with 1 mM HAuCl_4_ and NADH at 37 °C. AuNS were collected by centrifugation and washed 3 times with sterile water for 10 min at 5000x*g* and stored at 4 °C. AuNS were visualized by TEM.

### Data analysis

Statistical analysis and graphs were carried out using GraphPad Prism 6.0 (GraphPad Software, Inc.). The confidence interval in the analysis of variance (ANOVA) was set at p < 0.05. The statistical significance was indicated as follows: ∗p < 0.05, ∗∗p < 0.01, ∗∗∗p < 0.001 and ∗∗∗∗p < 0.0001; *ns* not significant.

## Supplementary information

**Additional file 1: Figure S1**. HAuCl_4_ susceptibility of *E. coli* grown under anaerobic conditions. **a***E. coli* growth anaerobically in the presence of the indicated Au^3+^ concentrations. **b** Relationship of the area under the curve (AUC) and HAuCl_4_ concentration.

**Additional file 2: Figure S2.** Viability of *E. coli* exposed to Au^3+^ with pretreatments of ROS scavengers. Cells grown to OD_600_ 0.4 were incubated for 30 min in the absence and presence of 2,2 bipyridyl and ascorbic acid, washed and incubated with 0.2 mM Au^3+^ for 15 min. The letters indicate the significance of the one-way statistical analysis ANOVA Multiple comparisons. ****p < 0.0001, **p < 0.05; *ns* not significant.

## Data Availability

All data generated or analyzed during this study are included in this published article.

## Abbreviations

AuNS: Nanostructures
ROS: Reactive oxygen species
MIC: Minimal inhibitory concentration
GSH: Glutathione
NS: Nanostructures
AUC: Area under the curve
SDS: Sodium dodecyl sulfate
ONPG: O-nitrophenyl-β-d-galactopyranoside
DHE: Dihydroethidine
RSH: Intracellular thiol
