# Supplementary material for: Understanding gold toxicity in aerobically-grown Escherichia coli
Source: Biol Res. 2020 Jun 8;53:26. doi: 10.1186/s40659-020-00292-5 (PMC7278051; doi:10.1186/s40659-020-00292-5)
Supplement: Supplementary file 1 — Additional file 1: Figure S1. HAuCl4 susceptibility of E. coli grown under anaerobic conditions. aE. coli growth anaerobically in the presence of the indicated Au3+ concentrations. b Relationship of the area under the curve (AUC) and HAuCl4 concentration. [file 40659_2020_292_MOESM1_ESM.docx]

Supplementary Material

**Understanding gold toxicity in aerobically-grown *Escherichia coli***

Muñoz-Villagrán C.^1,2^, Contreras F.^1^, Cornejo F.^1^, Figueroa M.^1^, Valenzuela D.^3^, Luraschi R.^1^, Reinoso C.^3^, Rivas-Pardo J.^1,4^, Vásquez C.^1^, Castro M.^3¶^ and Arenas F.^1¶^

**Correspondence to:** Felipe A. Arenas

E-mails: [felipe.arenass@usach.cl](mailto:felipe.arenass@usach.cl)


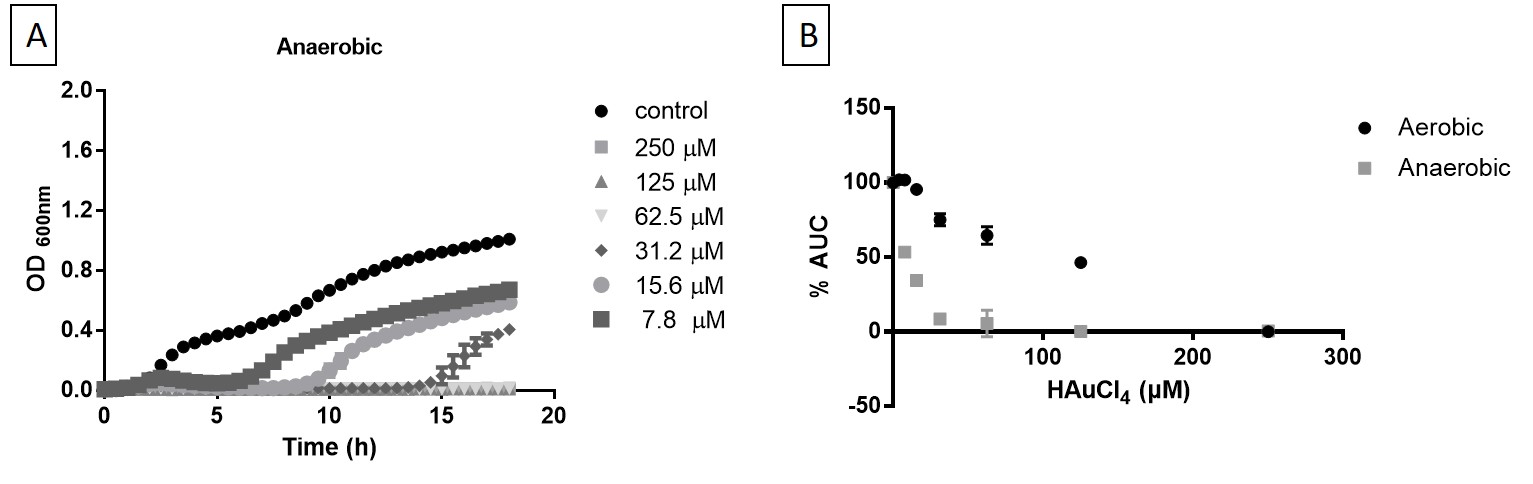


**S1 Fig. HAuCl_4_ susceptibility of *E. coli* grown under anaerobic conditions. (A)** *E. coli* growth anaerobically in the presence of the indicated Au^3+^ concentrations. (**B)** Relationship of the area under the curve (AUC) and HAuCl_4_ concentration.
