# Supplementary material for: Understanding gold toxicity in aerobically-grown Escherichia coli
Source: Biol Res. 2020 Jun 8;53:26. doi: 10.1186/s40659-020-00292-5 (PMC7278051; doi:10.1186/s40659-020-00292-5)
Supplement: Supplementary file 2 — Additional file 2: Figure S2. Viability of E. coli exposed to Au3+ with pretreatments of ROS scavengers. Cells grown to OD600 0.4 were incubated for 30 min in the absence and presence of 2,2 bipyridyl and ascorbic acid, washed and incubated with 0.2 mM Au3+ for 15 min. The letters indicate the significance of the one-way statistical analysis ANOVA Multiple comparisons. ****p < 0.0001, **p < 0.05; ns not significant. [file 40659_2020_292_MOESM2_ESM.docx]

Supplementary Material

**Understanding gold toxicity in aerobically-grown *Escherichia coli***

Muñoz-Villagrán C.^1,2^, Contreras F.^1^, Cornejo F.^1^, Figueroa M.^1^, Valenzuela D.^3^, Luraschi R.^1^, Reinoso C.^3^, Rivas-Pardo J.^1,4^, Vásquez C.^1^, Castro M.^3¶^ and Arenas F.^1¶^

**Correspondence to:** Felipe A. Arenas

E-mails: [felipe.arenass@usach.cl](mailto:felipe.arenass@usach.cl)


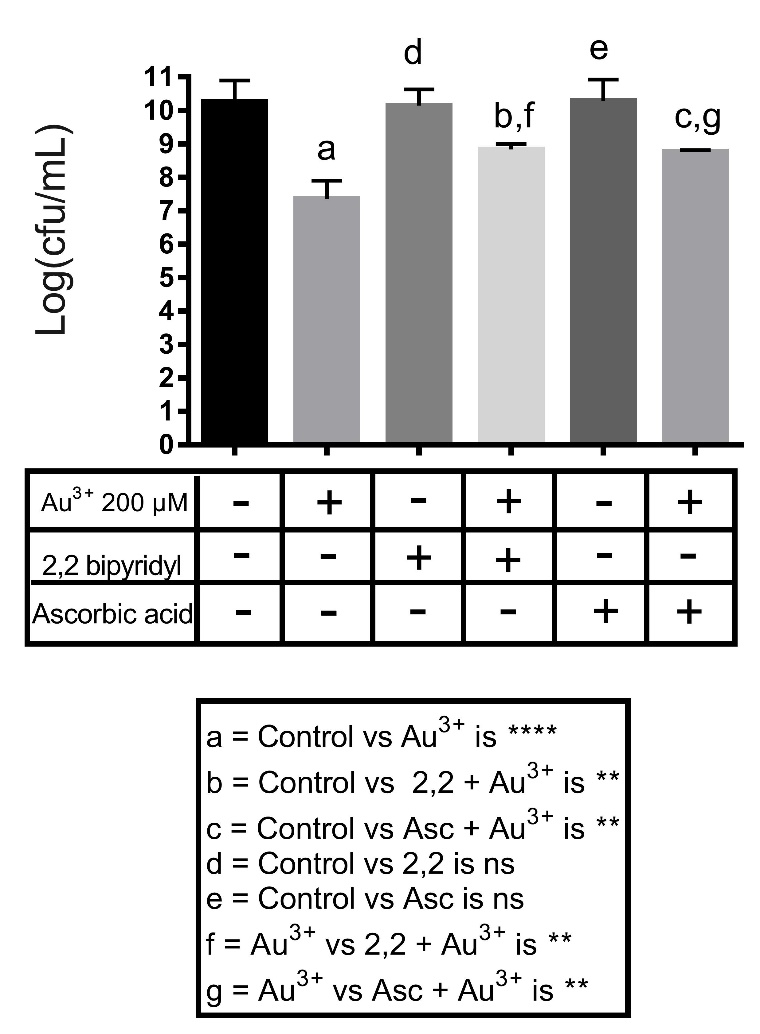


**S2 Fig. Viability of *E. coli* exposed to Au^3+^ with pretreatments of ROS scavengers.** Cells grown to OD_600_ 0.4 were incubated for 30 min in the absence and presence of 2,2 bipyridyl and ascorbic acid, washed and incubated with 0.2 mM Au^3+^ for 15 min. The letters indicate the significance of the one-way statistical analysis ANOVA Multiple comparisons. **** p <0.0001, ** p <0.05; ns, not significant.
